# Pregnancy‐induced progressive change of prolactin‐secreting macroadenoma with the development of bitemporal hemianopia and severe headache

**DOI:** 10.1002/ccr3.2244

**Published:** 2019-06-03

**Authors:** Sirinart Sirilert, Kuntharee Traisrisilp, Tawiwan Pantasri, Theera Tongsong

**Affiliations:** ^1^ Department of Obstetrics and Gynecology, Faculty of Medicine Chiang Mai University Chiang Mai Thailand

**Keywords:** macroadenoma, pregnancy, prolactinoma

## Abstract

In a difficult case of macroadenoma with progressive change during pregnancy, timely cesarean delivery, avoidance of breastfeeding, and intensive conservative treatment after birth could have satisfactory results, in terms of fetal outcomes, regression of tumor, and resumption of visual activity.

## 1 INTRODUCTION

A difficult case in conservative management of macroadenoma with progressive change during pregnancy and failure of medical treatment is described. Timely cesarean delivery, avoidance of breastfeeding, and intensive conservative treatment after birth could have satisfactory results in terms of fetal outcomes, regression of tumor, and resumption of visual activity.

Prolactinoma or lactotroph adenoma is a prolactin‐producing pituitary tumor, accounting for approximately 40% of all pituitary tumors. It could be asymptomatic or present clinical manifestations of hyperprolactinemia, such as abnormal menstruation, infertility, and galactorrhea. The Endocrine Society (2011)^1^ recommends that patients with symptomatic microadenoma or macroadenoma (larger than 1 cm in diameter) be treated with dopamine agonists. However, cabergoline is preferred to other dopamine agonists due to its higher efficacy in normalizing prolactin levels and reducing tumor size. Regarding prolactinomas with resistance to dopamine agonists, which do not achieve a significant reduction in tumor size or prolactin levels on standard dosage, it is recommended that the highest dosage that can be tolerated be used. Transsphenoidal surgical resection of tumors is rarely indicated but it may be considered in cases of medical failure. Finally, in patients with medical or surgical failure, radiation therapy is suggested.

In normal pregnancy, pituitary gland volume is increased by about 70%. Accordingly, pituitary adenomas tend to be enlarged during pregnancy, and they cause symptoms such as headache and visual loss. Pregnancy can cause tumor growth, infarction, and hemorrhage. Though both microadenoma and macroadenoma can be progressive and cause symptoms, macroadenoma leads to a much more significant change. Nevertheless, the growth of prolactinoma during pregnancy, which causes pressure effect on the optic chiasm, leading to visual loss, has been reported only a limited number of times. Additionally, because of its rarity, the most appropriate management for such growing tumors with medical failure is still very challenging, especially in terms of delivery timing and route, surgical or radiation therapy during pregnancy, proper time for surgical delay after birth to wait for spontaneous resolution and medication. Therefore, we report a case of pregnancy‐induced progressive change of prolactin‐secreting macroadenoma with the development of bitemporal hemianopia and severe headache to increase the number of cases in the existing body of knowledge for the purpose of achieving future analysis with a larger sample size.

## 2 CASE

A 32‐year‐old pregnant woman (G2, P0010), with a pituitary prolactin‐producing macroadenoma (larger than 1 cm) was managed at Maharaj Nakorn Chiang Mai Hospital, Chiang Mai, Thailand. She had a history of miscarriage. The macroadenoma was diagnosed 1 year prior to the current pregnancy, based on a history of amenorrhea for 2 years, prolactin levels of 244.3 ng/mL, and the findings of the first CT brain scan, which showed the enlarged pituitary gland (1.6 × 1.2 × 1.3 cm), as well as the thin and sloping sella floor. Nevertheless, no other symptoms and abnormal physical or laboratory findings were observed.

**Figure 1:**
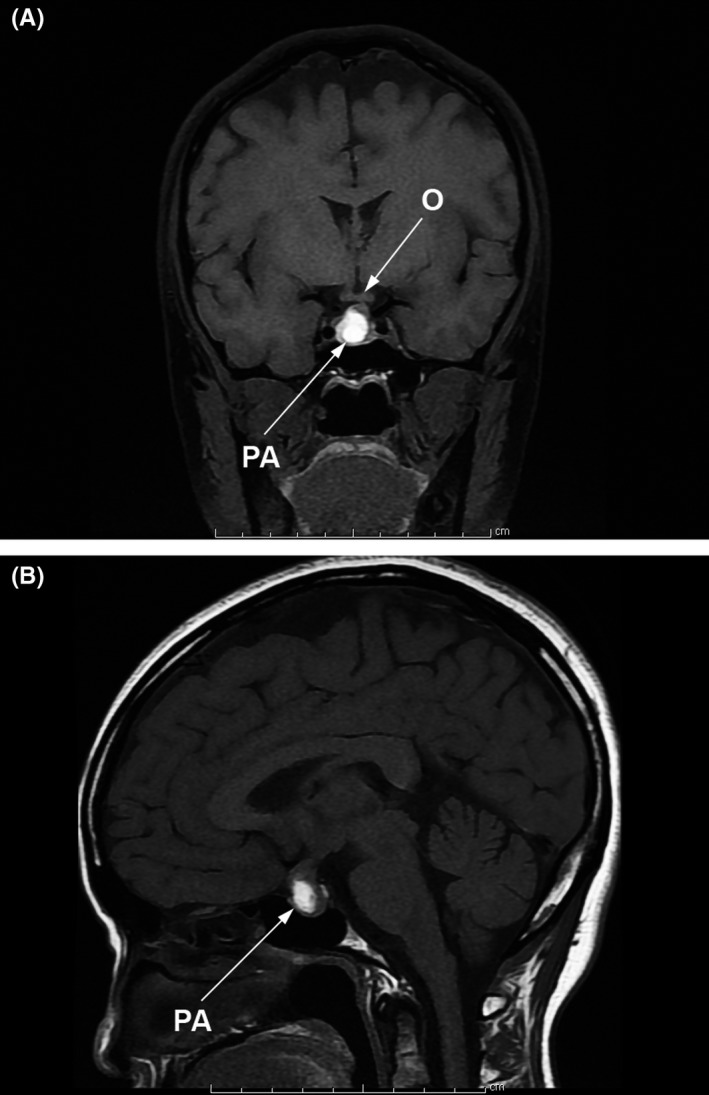
T1‐MRI Pituitary coronal (A) and sagittal (B) scan at 16 wk of gestation depicting pituitary apoplexy (PA) and minimal compression of optic chiasm (O)

After bromocriptine treatment, she got pregnant when her prolactin level was 19.74 ng/mL and other laboratory tests were normal. However, her baseline brain MRI when pregnancy was diagnosed (16 weeks of gestation) showed deviation of the pituitary stalk to the left side, enlarged anterior lobe of the pituitary gland (0.9 × 0.8 × 1.2 cm), and subacute hemorrhage (pituitary apoplexy) with minimal indentation of the optic chiasm (Figure 1). Bromocriptine therapy was continued throughout pregnancy. Clinical assessment was done at least once a month, and visual fields were tested every trimester and when indicated by clinical changes.

**Figure 2:**
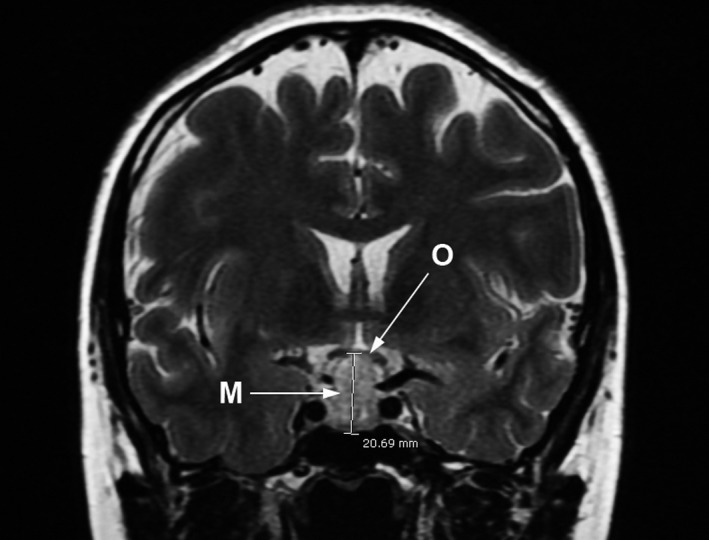
T2‐MRI Pituitary coronal scan at 32 wk of gestation: macroadenoma (M) with significant compression of optic chiasm (O)

She was admitted to the hospital at 32 weeks of gestation since she developed a visual disturbance. The brain MRI showed an increase in the size of the tumor to 2.0 × 1.6 × 1.5 cm with pressure effect, resulting in an upward displacement of the prechiasmatic segment of the bilateral optic nerves and the optic chiasm (Figure 2). Pituitary apoplexy was suspected. Hypothyroxinemia (FT4:0.89 ng/dL; TSH: 14 mU/L; anti‐TPO: negative) was also detected and treated with levothyroxine (50 mcg/d). However, the visual field testing showed normal results and no other neurological or surgical sign was observed. Therefore, the patient was conservatively managed with adjusted dosage of bromocriptine and levothyroxine. She was discharged when clinical improvement was observed. Nevertheless, she was admitted again at 37 weeks of gestation due to blurred vision with severe headache. The visual field testing showed bitemporal hemianopia. Urgent delivery was considered to avoid the progressive change of the tumor, and the route of delivery was discussed. The advantages and disadvantages of each route of delivery were explained to the patient and her family. Cesarean section was chosen by the patient to avoid intrapartum stress and possible further impairment of visual ability. An uneventful cesarean section was performed, giving birth to a female newborn, weighing 2920 grams with APGAR scores of 8 and 9 at 1 and 5 minutes, respectively.

**Figure 3:**
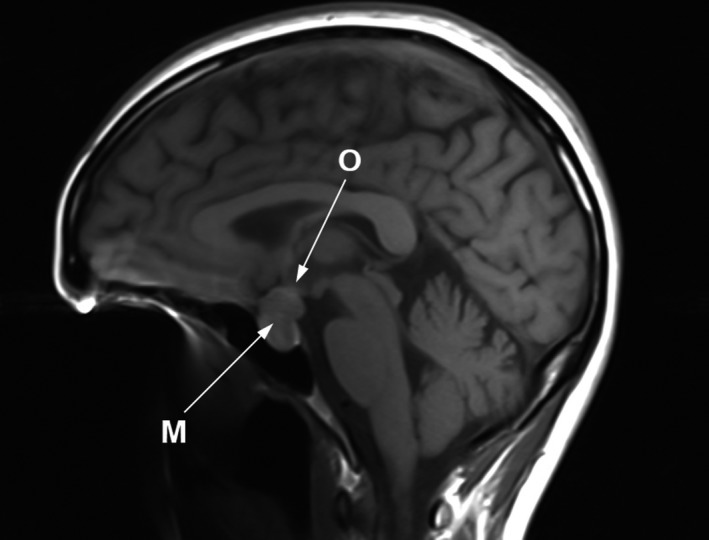
T1‐MRI Pituitary sagittal scan at 2 wk postpartum: macroadenoma (M) with significant compression of optic chiasm (O)

Breastfeeding was avoided and bromocriptine treatment was continued. Two weeks after birth, the abnormal visual field remained the same, and the brain MRI findings showed no significant change. The tumor size was relatively the same (1.5 × 1.6 × 2.0 cm) with suprasellar extension (Figure 3). The patient was scheduled for re‐evaluation at 1 month after birth to give a chance for tumor regression, and transsphenoidal surgery would be considered in case of no medical improvement. At 1 month postpartum, the prolactin level reduced from 1459 ng/mL at 2 days postpartum to 427 ng/mL. During that period, the visual field also gradually improved significantly.

The improvement of the visual field and the decreasing prolactin levels indicated that bromocriptine therapy gave a satisfactory response after 1 month postpartum. Accordingly, there was no indication for an operation. She was continuously treated with bromocriptine and levothyroxine with no side effect.

## 3 DISCUSSION

During pregnancy, the pituitary gland may be physiologically enlarged, and prolactin levels are increased. Thus, prolactin level measurements and routine pituitary MRI are not recommended during pregnancy. However, pituitary MRI without gadolinium is recommended for patients with worsening symptoms or abnormal visual field testing. Regarding the literature review by Molitch,^2^ the rates of symptomatic tumor growth were 2.4% for microadenoma, as much as 21% for macroadenoma, and 4.7% for macroadenoma with prior debulking surgery or radiation. Accordingly, women with microadenoma should be advised to discontinue dopamine agonist therapy, but selected patients with macroadenoma who have no previous surgical or radiation therapy may prudently continue dopaminergic therapy throughout the pregnancy.^1^

However, pregnancy can induce remission of hyperprolactinemia by an uncertain mechanism, presumably secondary to autoinfarction of the tumors.^3^, ^4^ Additionally, a patient with mass effect presented as acute diplopia and ptosis in the antepartum period was reported to have a complete resolution without any surgery at 5 months after delivery.^5^ Interestingly, the case presented here showed obvious pituitary apoplexy (Figure 1), but the tumor further increased and caused symptoms. Breastfeeding may or may not be avoided. There are limited data suggesting the association between breastfeeding and an increased prolactin level or tumor enlargement.^4^, ^6^ Based on the above review, the prognosis and proper management of macroadenoma during pregnancy is still uncertain.

The management in our case followed the Endocrine Society’s recommendation by continuing bromocriptine treatment. However, continuous use of bromocriptine throughout pregnancy has been reported in only a limited number of patients.^7^ The results showed that it does not cause adverse pregnancy event or fetal malformation. We did not measure prolactin level and routine follow‐up MRI. The symptomatic tumor growth of macroadenoma during pregnancy with viable preterm fetus, like the case presented here, is challenging. With this condition, management is not clearly recommended.^1^ In general, for term or nearly term fetuses, induction of labor before neurosurgical intervention may be reasonable. However, for previable fetuses, management is complicated; there are no published data to access the comparative risk of continuing bromocriptine treatment and surgical resection during pregnancy, though surgical resection during pregnancy in some selected cases was reported to be safe.^8^, ^9^, ^10^ In our case, visual field tested with Goldmann perimetry technique revealed normal results and needed no surgical treatment for enlarged pituitary gland during early pregnancy.

Though dopamine agonist therapy should be suspended until cessation of lactation for a woman who desires to breastfeed, in severe cases like our patient, dopamine agonist therapy might reasonably be continued and breastfeeding might be avoided to theoretically avoid acute and rapid progression of the disease. Though we had initially planned to perform surgery after postpartum failure of medical treatment because of severe headache and to prevent further visual impairment, a challenging delay was instituted with close observation. Gradual improvement was observed after 2 weeks postpartum and finally, surgery could be safely avoided. Therefore, our case supports the assertion that the delay of surgical treatment may be reasonable in the postpartum period.

Unlike microadenoma or asymptomatic macroadenoma during pregnancy, which have been reported several times, pregnancy‐induced worsening of macroadenoma, causing bilateral hemianopia with severe headaches, is rarely reported. Accordingly, the management guideline for cases of asymptomatic macroadenoma during pregnancy with progression to become symptomatic and with an increase in size in spite of dopamine agonist control is still very challenging, especially in terms of delivery timing and route, surgical or radiation therapy during pregnancy, and proper time for surgical delay after birth to wait for spontaneous resolution and medication. Our case emphasizes that pregnancy‐induced growth of macroadenoma can lead to symptomatic mass effects and management is very challenging and individualized. This case is an example of difficulty in making decision. At this point, two main questions must be raised: (a) In a situation of growing symptomatic mass at a time that is far from term, should the pregnancy be delivered or not? (b) Would the symptomatic mass effects spontaneously resolve after pregnancy termination in all cases or not and when should surgical approach be offered? However, our case provides evidence that symptomatic pregnancy‐induced growth of macroadenoma may be conservatively managed, and surgery after birth may be delayed at least 1 month with close follow‐up.

In cases of no obstetric indications for cesarean section, vaginal delivery can be safely performed in women with microadenoma. Nevertheless, no data on the safety of vaginal delivery for macroadenoma with visual impairment or severe headache like our case have been reported, and decision on the route of delivery is individualized. Vaginal delivery in cases of asymptomatic macroadenoma may possibly be justified; however, in cases of symptomatic macroadenoma, we do not know whether or not the alteration of brain circulation especially in the pituitary gland, during the several hours of labor as well as mental and physical stress can affect the symptoms, especially visual impairment. Also, we do not know whether or not they increase the risk of pituitary hemorrhage or apoplexy. From our point of view, symptomatic macroadenoma should be an indication for cesarean section until more data on the safety of vaginal delivery have been confirmed.

Based on most previous studies, breastfeeding is not associated with an increased prolactin production or risk of tumor enlargement.^4^, ^6^, ^11^ Thus, women can safely breastfeed and restart dopamine agonist therapy after cessation of lactation. However, previous reports are based on patients with microadenoma and asymptomatic macroadeonoma. No data support the safety of breastfeeding and dopamine agonist discontinuation among patients with symptomatic macroadenoma. Nevertheless, breastfeeding certainly stimulates lactotroph cells to increase prolactin production. Therefore, macroadenoma with visual impairment, like our case, may theoretically be at risk of tumor stimulation by breastfeeding and cessation of dopamine agonists during the postpartum period. Accordingly, breastfeeding should be avoided and dopamine agonists should be continued during the postpartum period in symptomatic macroadenoma.

## 4 CONCLUSION

We reported a case of macroadenoma with progressive change, including pituitary apoplexy and further enlargement of mass with pressure effect on the optic chiasm leading to visual field defects and persistent headache. Because of the failure of medical treatment, we performed cesarean delivery. Also, we implemented a 1 month postpartum delay before considering surgery, resulting in regression of the tumor to the pre‐pregnancy size and the disappearance of all symptoms. This case suggests that timely cesarean delivery, avoidance of breastfeeding, and intensive conservative treatment after birth in some selective cases could have satisfactory results, in terms of fetal outcomes, regression of the tumor, and resumption of visual activity.

## CONFLICT OF INTERESTS

None declared.

## AUTHOR CONTRIBUTIONS

SS: involved in obstetric care of the patient and medical review/record, and manuscript preparation; KT: involved in obstetric care of the patient and medical review/record; TP: involved in endocrinological care of the patient; TT: involved in medical review/record and manuscript preparation.

## PATIENT CONSENT

Obtained.
